# Electrophysiological correlates of top-down attentional modulation in olfaction

**DOI:** 10.1038/s41598-019-41319-6

**Published:** 2019-03-20

**Authors:** Archana K. Singh, Kazushige Touhara, Masako Okamoto

**Affiliations:** 1 Department of Applied Biological Chemistry, Graduate School of Agricultural and Life Sciences, The University of Tokyo, Tokyo, 113-8657 Japan; 2 ERATO Touhara Chemosensory Signal Project, JST, The University of Tokyo, Tokyo, Japan; 3 International Research Center for Neurointelligence (WPI-IRCN), The University of Tokyo Institutes for Advanced Study, Tokyo, Japan

## Abstract

The capacity to pay attention is important for the cognitive ability, for example, evaluating an object for its qualities. Attention can selectively prioritize the neural processes that are relevant to a given task. Neuroimaging investigations on human attention are primarily focused on vision to the exclusion of other sensory systems, particularly olfaction. Neural underpinnings of human olfactory attention are still not clearly understood. Here, we combined electroencephalographic measurements of olfactory event related potential with electrical neuroimaging to investigate how the neural responses after inhaling the same odor differ between conditions with varying levels of attention, and, in which brain areas. We examined the neural responses when participants attended to a rose-like odor of phenylethyl alcohol for evaluating its pleasantness versus its passive inhalation. Our results gathered significant evidence for attentional modulation of the olfactory neural response. The most prominent effect was found for the late positive component, P3, of olfactory event related potential within a second from the odor onset. The source reconstruction of this data revealed activations in a distributed network of brain regions predominantly in inferior frontal cortex, insula, and  inferior temporal gyrus. These results suggest that the neuronal modulations from attention to olfactory pleasantness may be subserved by this network.

## Introduction

Attention is the ability to choose and concentrate on relevant stimuli, and has been a topic of active research interest among neuroscientists. However, much of the neuroimaging literature on top-down control of attention is centered on vision. In comparison, the research related to olfactory attention is relatively new and still emerging. Study of attention has a specific relevance to human olfaction, because we often do not pay attention to our olfactory surroundings, and remain oblivious to the odors around us^1,2^. How we perceive odors depends on how our attention is focused to our olfactory environment. Attentional focus can be driven by bottom-up (exogenous) factors, for instance, a salient odor will involuntarily draw our attention. In contrast, top-down attention is driven by endogenous factors. Sometimes, we volitionally focus our attention to selected olfactory aspects, e.g., to rate its pleasantness or intensity, in an odor evaluation task. Such selective prioritization manifests in improved task performance. For example, attending to an odor for evaluating its intensity can evoke a faster response time relative to when attention is directed elsewhere^3^.

Such task-related top-down control of attention is also known to enhance neural activity in the brain areas that underlie neural processing of the attended stimulus. Previous olfactory event related potential (OERP) studies have shown that attending to the olfactory stimulus for its active evaluation, e.g., in an intensity judgement or detection task, results in greater amplitude of its later peak, P3, when compared to its passive inhalation in a non-olfactory task or a relax condition^4–7^. Among OERP components, P3 is the most distinguishable peak, which appears in post-stimulus time-intervals of 700–1300 ms. Others reported shorter ERP latencies when olfactory stimuli are attended^6,8^. A couple of these studies suggested that top-down manipulation of attention can also influence early OERP components, N1 and P2 in the respective post-stimulus time-intervals of 320–500 ms, and 550–700 ms^5,6^. Expecting to receive a potentially hazardous or painful odor can also lead to shorter latency of early peaks^9,10^.

In the context of examining task-related attentional modulation on OERP, larger P3 amplitude for a given odor implies that greater amount of neuronal resources are recruited for processing it in the context of attend than not-attend task; the latency of P3 (that is, the time at which peak reaches its maxima) signifies the timing of processes underlying stimulus evaluation^11^. It is worth noting here that the term *attentional modulation* in neuroimaging literature is commonly used to refer to task-related differences in neural activity, which may also be due to other cognitive factors that closely interact with attention, e.g., memory and decision making. Therefore the attentional modulation in neural activity, e.g., increased P3 amplitude in attended (than not attended) odor condition, should reflect all of these.

The existing fMRI and PET literature suggest the involvement of primary olfactory areas (e.g., piriform cortex) as well as secondary olfactory areas (e.g., orbitofrontal cortex) in various odor processing tasks; see for reviews^12^ and^13^. In addition, it is widely believed that olfactory sensory neurons are projected to the neocortical brain regions without a direct (or a weak) involvement of thalamus^1,14,15^, and it is proposed that olfactory bulb, where the first synapse of sensory neuron occurs, may have an important role in top-down control of selective attention^1^. Although neuroimaging investigations to identify cortical areas that specifically mediate task-driven olfactory attention are scant, an emerging view from their findings suggests that olfactory attention is mediated by a dynamic network of brain areas depending on the task involved. For example, orbitofrontal gyrus was found to be more activated when subjects attended to the odor to evaluate its pleasantness than intensity in an fMRI study^16^. A PET study reported increased activation in orbitofrontal cortex during both pleasantness and intensity evaluation tasks when compared with passive no-odor task^17^. An fMRI study that examined attention to hedonic properties versus passive inhalation of odor reported activation in orbitofrontal cortex, insula, and middle temporal gyrus^18^. Another fMRI finding relevant to olfactory attention comes from a study that compared an odor detection task with the passive inhalation of a blank odorless stimulus, and reported activation in piriform cortex, orbitofrontal gyrus, amygdala, entorhinal cortex, insula and superior temporal gyrus^19^.

While fMRI provides excellent spatial resolution, its temporal resolution is limited to the order of seconds. This is too slow for capturing the inherently transient neural responses to an attended odor stimulus that change in the order of milliseconds. Therefore, fMRI can show the network of brain areas involved in an experimental task, but the information about time course of their activation is lost. More precise information on the time course of neural activity is provided by event related potentials (ERPs) measured with Electroencephalography (EEG).

However, the evidence on olfactory attention from OERP studies (as referenced above) thus far is limited to only three midline electrodes, Fz, Cz, and Pz. The neuronal generators underlying such modulatory effect of olfactory attention on OERP components are still not known. A complete picture of the timing, spatial pattern, and localization of these effects can provide a better basis for delineating the neural substrates of olfactory attention. A higher-dimensional EEG probe that covers the entire scalp can afford a more spatially informed inference, e.g., about how attention modulates the scalp topography of P3 OERP component. It has long been speculated that the topographic patterns of OERP amplitudes can provide a putative link between the scalp recorded OERP and its underlying neuronal generators^20,21^. In addition, high-dimensional EEG permits the use of electrical neuroimaging, which is based on robust algorithms, to estimate the neuronal sources from the distribution of electrical activity on the scalp^22,23^. This offers the unique advantage of localizing the sources of ERP measurements in the Montreal Neurological Institute (MNI) brain template, albeit with a coarser spatial resolution than fMRI, at specific time points and stages of processing at the resolution of milliseconds. The application of source reconstruction methods to olfactory ERP has recently been explored though for tasks not related to attention^24,25^.

In this study, we re-examined electroencephalographic correlates of olfactory attention with these contemporary methods. We used higher-dimensional 64-channel EEG measurements to examine attentional modulation in N1, P2, and P3 OERP peaks, and their scalp topography patterns. In addition, we estimated the cortical sources underlying the observed effects of top-down attentional modulation in olfaction. Similar to previous studies on olfactory attention, our experiment design involves repeated presentation of odor stimuli such that some trials require participants to evaluate the odor and others do not^4–7^. In our experiment, participants were required to evaluate the pleasantness of odor during attend (AT) trials, and not-attend (NA) trials comprised passive inhalation of the same odor. The purpose of including pleasantness rating task was to manipulate top-down attention. Since we used the identical odor between attend and not-attend task, the design allowed us to examine the differences in their cortical processing in terms of top-down attentional modulation.

## Material and Methods

### Participants

Thirty Eight subjects participated in this study (age 23.6 ± 4.4 years (Mean ± SD), 15 women). They were recruited by advertising in the campus of University of Tokyo. Only the healthy participants who had no respiratory and nasal problems, and had a self-reported normal sense of smell were asked to participate. In addition, we also conducted *Open Essence test*, which allows odor identification assessment using odors familiar to Japanese people^26^. Similar to the scores reported in this study for participants with normal olfactory ability, our scores ranged from 7 to 12 with a median of 10 (Supplementary Table S1). The participants were instructed to refrain from cosmetics, food and drinks with strong smell, smoking and alcohol on the day of experiment. The study was approved by the ethics committee of University of Tokyo and in accordance with the Declaration of Helsinki. The experiments were conducted after obtaining the written informed consent from the participants, who received a modest participation fee.

### Olfactory Stimuli and Presentation

We used Phenylethyl alcohol (PEA, a rose-like smell) as the odor stimulus, and H_2_O as the odorless control. In addition, we used Vanillin (VAN) in filler trials. PEA was perceived to be neutrally valenced, and VAN as pleasant in a pilot screening conducted with a separate group of participants (n = 10, 5 women, age 22–28 years). If only PEA stimulus was used across all odor trials, participants could easily guess it, and predict its pleasantness without paying much attention. The VAN odor was included in filler trials to control the predictability of the odor stimulus of interest (PEA), which were removed from ERP analysis. The odorants were diluted in polypropylene glycol - PEA (15% vol/vol) and VAN (5% wt/vol). At the selected concentrations, they are unlikely to stimulate trigeminal nerve^27^.

Odor stimuli were presented in a computer controlled set up using olfactometer and breathing sensor (Burghart Instrument, Wedel, Germany). The olfactometer provides a constant flow of humidified air (base airflow) via a tube to the nose without altering the mechanical conditions of mucosa using air-dilution principle^28^. During the stimulation duration, the olfactometer switches the base airflow with the stimulus (odor or odorless) air. The air for each stimulus was generated from a separate liquid jar. The humidifying jar for the base airflow and liquid jar for the control stimulus contained the same odorless water, H_2_O. The participants inhaled the odor through a small cylindrical nose-piece inserted approximately 5 mm into the right nostril. The odor onset time was synchronized with the subject’s inspiration by means of breathing sensor, which can detect inhalation onset by sensing pressure variations at the nose-piece. This allowed participants to maintain natural breathing nasally throughout the experiment and avoid active sniffing during the stimulus presentation. The stimulus duration was set as 500 ms for all the stimuli (the rise time was not more than 60 ms according to the maker specifications).

The olfactometer was located outside the electrically shielded EEG chamber with a consistent ambient atmosphere, at room temperature of 24.7 ± 1.1 °C (Mean ± SD) and relative humidity of 47.7 ± 7.4% (Mean ± SD), as recorded over experiment sessions for the 38 subjects who participated in the study. Participants were seated in a relaxed position, and used earplugs to mask valve switching click sounds from the olfactometer. Instructions and cues for the experiments were shown using a computer screen placed 80 cm away from the participant. The stimulus presentation was tested for the required conditions in an experiment with a separate group of six trained subjects.

### Conditions

**Figure 1:**
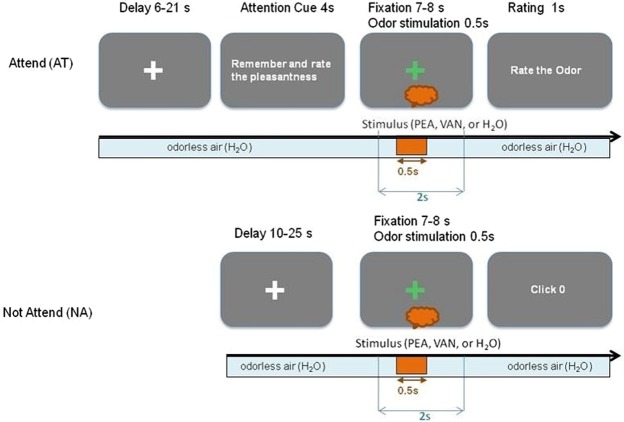
Experiment Design. The timing of events are shown for trial types AT (top panel) and NA (bottom panel). A baseflow of odorless air (H_2_O) was used throughout the experiment except during olfactory stimulus presentation. For each condition, three stimuli, experimental odor (PEA), odorless control (H_2_O) or filler odor (VAN), denoted as orange bubble, was presented within the fixation period. The fixation phase lasted 7–8 s. The order of the stimulus presentation was systematically randomized so that we could yield 5 trials per condition for PEA and H_2_O, and 2 trials per condition for VAN in each session. The stimulus duration was 500 ms as indicated by orange arrow. The stimulus onset was jittered and presented after 3–5 s from fixation phase onset. The epochs for ERP data were extracted from the peri-stimulus interval of 2 s (from 500 ms before to 1500 ms after the stimulus onset) as marked in blue arrow. There were six sessions in each experiment.

There were two trial types: attend (AT), which required participants to attend to the pleasantness of smell from the air that they inhaled, and not-attend (NA), which did not require this attention (Fig. 1). In both cases, PEA, H_2_O, or VAN stimulus was presented. The experimental conditions of interest were defined by labeling the pairs of stimuli (PEA, H_2_O) and trial types (AT, NA) as follows: PEA-AT (PAT), PEA-NA (PNA), H_2_O-AT (HAT), and H_2_O-NA (HNA). The filler conditions, VAN-AT (VAT), VAN-NA (VNA) were omitted from the analysis.

### Procedure

Each AT trial commenced with a delay phase (6–21 s) when participants focused on a white cross (Fig. 1, top panel). Then, during the cue phase, participants were instructed to remember and rate the odor pleasantness using a visual cue (4 s), followed by a fixation phase where participants focused on a green cross (7–8 s). The participants were asked to strictly avoid blinking in this period and pay attention to the odor. The olfactory stimulus was presented within this fixation phase. The stimulus onset time was jittered in an interval of 3–5 s from fixation onset and stimulus duration was 500 ms (Fig. 1). In the response phase, participants rated the odor as ‘Unpleasant’, ‘Neutral’, ‘Pleasant’ or ‘No-smell’ by pressing specific keys on the keyboard, labeled as 1, 2, 3 and 0, respectively. The no-smell rating was for indicating when they did not perceive any odor. Prior to the experiment, participants were informed as follows. When they received an attention cue, they should remember the smell of the air. If the air smells, they should rate its pleasantness, as unpleasant, neutral, or pleasant. If it did not smell, they should rate it as no-smell. As expected on the basis of results from pilot odor screening test, most frequent rating was ‘Neutral’ for PEA, ‘Pleasant’ for VAN, and ‘No smell’ for H_2_O (Supplementary Fig. S1).

In NA trial, there was no cue phase (Fig. 1, bottom panel). The delay phase (10–25 s) was followed by the fixation phase, which was identical in design to that for AT trial except that here participants were asked not to pay any attention to the odor of the air. The trial ended with a trivial response from the participants by pressing a key to proceed the experiment.

The inter-stimulus-interval (ISI) for both trial types was 15–45 s and two identical odor stimuli were separated by at least 30 s to avoid adaptation. The six experimental conditions were presented in a pseudo-random order across 30 trials in each session so that we could acquire 5 trials each for PAT, PNA, HAT, and HNA, and 2 trials each for VAT and VNA. Each subject performed 6 experiment sessions. Thus each participant received 144 trials per experiment.

The jittered odor onset during fixation phase and variable ISI across trials were used to minimize odor related expectancy. The participants knew that odorous or odorless air would be presented sometime during the fixation period, but they had no information on what and how many stimuli were used in each session. They also did not know how many trials and which trials were odorless. An experiment session lasted about 8–13 min. There was a rest break for 3–5 min between sessions. Subjects were given explanation and training before the experiment for 15–20 min.

Our experiment design followed a single-stimulus paradigm as used in previous studies^5,8^. The purpose of odor pleasantness rating was to manipulate different levels of attention between AT and NA trial types using single identical odor. We used this paradigm with a *delayed-response* option such that the participants could evaluate the odor for a few seconds before responding^29^. This allowed us to extract the neural response during trials without any motor-related confounds. The response phase was not included in ERP data.

### EEG recording

Electroencephalographic signals were obtained from 64 scalp electrodes according to the international 10–20 system using Biosemi Active Ag-AgCL electrodes. The electrodes were mounted using gel through the holders contained in the Biosemi head cap. The electro-oculogram (EOG) was recorded from electrodes placed at the outer canthi of both eyes and below and above the left eye. The data from two mastoid electrodes was included for later offline referencing. The signals from all electrodes were digitized at a rate of 512 Hz.

### EEG preprocessing

In Biosemi EEG recording, online low pass filtering is performed in the ADC’s (analog-to-digital converter) decimation filter (hardware bandwidth limit), which has a 5th order sinc response with a −3 dB point at 1/5th of the selected sample rate. In addition, we applied an offline high-pass filter at 0.05 Hz cut-off frequency.

The continuous time series data for each electrode was inspected for noise using semi-automatic procedures. The continuous recording was divided into 2000 ms epochs for each trial, beginning 500 ms before the stimulus onset. Independent component analysis (ICA) was performed on epoched data to identify and correct artifacts related to eye movement based on the component topography and power spectrum. After ICA, we applied additional low-pass filter at 20 Hz cut-off. The filtering was performed using finite impulse response (FIR) option in EEGLAB, that applies the filter forward and then backward to ensure that the phase delays introduced by filtering are nullified^30^. The trials in which absolute amplitude exceeded 100 *μ*V in the vertical EOG (top minus bottom vEOG), or 80 *μ*V at any scalp electrode and time point in the epoch interval of 2 s were rejected, to remove any remaining paroxysmal, eye-blink, movement and muscle artifacts. The data for noisy electrodes were interpolated. The data for two subjects were removed because of hardware defect. The data for five more subjects were removed because they had difficulty in fixating, and had eye-blink related noise in almost all the trials. The final analysis comprised of clean epoched data from 31 subjects (age 23.7 ± 5.1 years (Mean ± SD), 12 women). The average number of trials (Mean ± SD) that remained after artifacts correction procedure were 27 ± 2 per condition.

All data preprocessing and analyses were done with custom written MATLAB scripts and EEGLAB, an open source toolbox for EEG data analysis^30^.

### OERP Analysis

The ERPs were extracted from EEG epochs after baseline correction with reference to a pre-stimulus baseline of −500 ms to 0 ms, and averaged across participants to visualize the waveforms for PAT, PNA, HAT and HNA conditions. The time windows for identifying N1, P2 and P3 peaks were set as 320–500 ms, 450–700 ms, and 700–1300 ms respectively, following previous OERP studies^5–7,31–33^. The mean peak amplitudes were obtained for each subject by averaging the data over time intervals around the observed peaks in the grand-averaged waveform. The component latencies were determined as the time of maximum amplitude within their specified time intervals for each subject.

Using non-parametric paired bootstrap t-test (with 10000 replications), we first examined the topographic patterns of OERP components to obtain group-level significance of mean amplitude and latency difference between PAT and PNA. The p-values were determined from the distribution of the t-statistic, which was obtained from surrogate bootstrap samples using 10000 replications. The resulting p-values were corrected for multiple comparison using positive false discovery rate controlling procedure, pFDR^34^. This method provides an estimate of the false discovery rate, which is determined empirically from the null distribution after setting the tuning parameter lambda to a range [0.01:0.01: 0.95]. The significance of pFDR is measured as *q-value*.

For verification, we compared the amplitude topographies of odor ERP with odorless ERP components using the same procedures.

### Source localization analysis

Source localization was performed using Standardized low resolution brain electromagnetic tomography (sLORETA) software package by^35^ (http://www.uzh.ch/keyinst/loreta.htm) for OERP components that revealed significantly greater amplitude for AT than NA trials. This method allows a robust estimation of the cortical sources of the neural activity in a specific time window. It follows a distributed source localization algorithm with minimum norm approach to solve the inverse problem of brain electric activity, which assumes that electric potentials are generated by a large number of dipolar sources distributed on the cortical surface, and so it does not require a priori knowledge of the number of neural generators^22,35^. This generates a 3D distribution of current source density for 6239 voxels with a spatial resolution of 5 mm, in an intracerebral volume, which comprises cortical gray matter and the hippocampus. The minimum norm solution is then normalized with its own standard deviation to improve localization accuracy specifically of the deeper cortical sources. In sLORETA, a realistic 3-shell boundary element method (BEM) head model from an averaged magnetic resonance image (MRI) dataset^36^ is used to represent the geometry of the brain, skull and scalp in MNI (Montreal Neurological Institute) brain template^37^. The cortical gray matter is determined based on probabilistic Talairach atlas^38^. The sLORETA toolbox generates a list of MNI co-ordinates, anatomical labels, and current density for the entire brain volume of 6239 voxels, which was used to identify voxels with significant brain activation. sLORETA is commonly used for localizing cognitive ERP components across sensory modalities^39–44^. The validation of this distributed source localization technique has been independently replicated by two studies^45,46^. Several studies have shown cross-modal validation combining LORETA with functional MRI (fMRI)^47,48^, and PET^49,50^.

The sLORETA current densities were computed from trial-averaged ERPs for 31 subjects. For the statistical analysis, mean sLORETA values were extracted for AT and NA trial types of all subjects, by averaging cortical current source densities in the same time interval of the ERP component as used in the OERP analysis. A non-parametric paired bootstrap t-test was performed on these values in a mass-univariate manner for all 6239 voxels, and pFDR method was applied for controlling false positive in the resulting inference.

The main purpose of this analysis was to identify brain regions that showed significantly greater current density for AT than NA for odor stimulus. In addition, we performed the same analysis with the odorless control stimuli for verification. We expected that for a given identical experimental task, the odor stimulus would elicit significantly greater OERP effect than control.

## Results

### OERP Analysis

**Figure 2:**
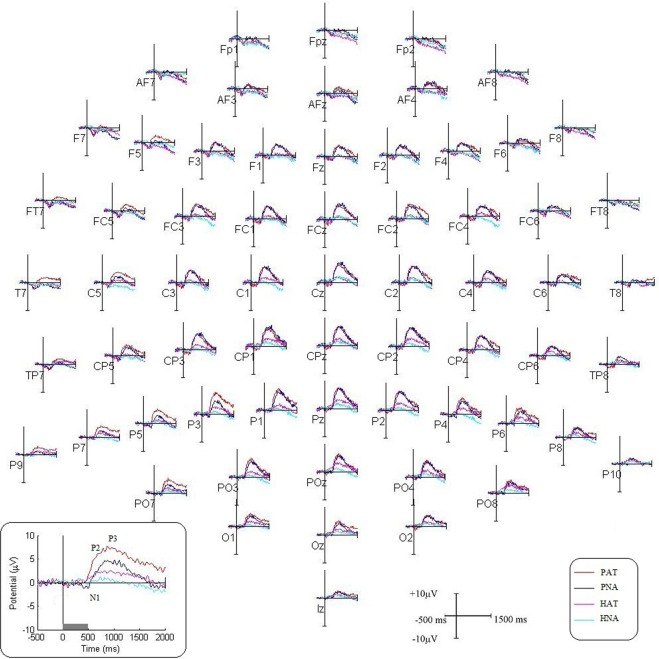
Comparison of odor and odorless ERP waveforms. The grand-averaged ERP plots for PAT (PEA-AT), PNA (PEA-NA), HAT (H_2_O-AT), and HNA (H_2_O-NA) conditions are shown for 64 electrodes. Inset box shows the magnified view of P3 electrode. The stimulus onset is marked by vertical line (0 s), and duration by grey bar (0–500 ms).

**Figure 3:**
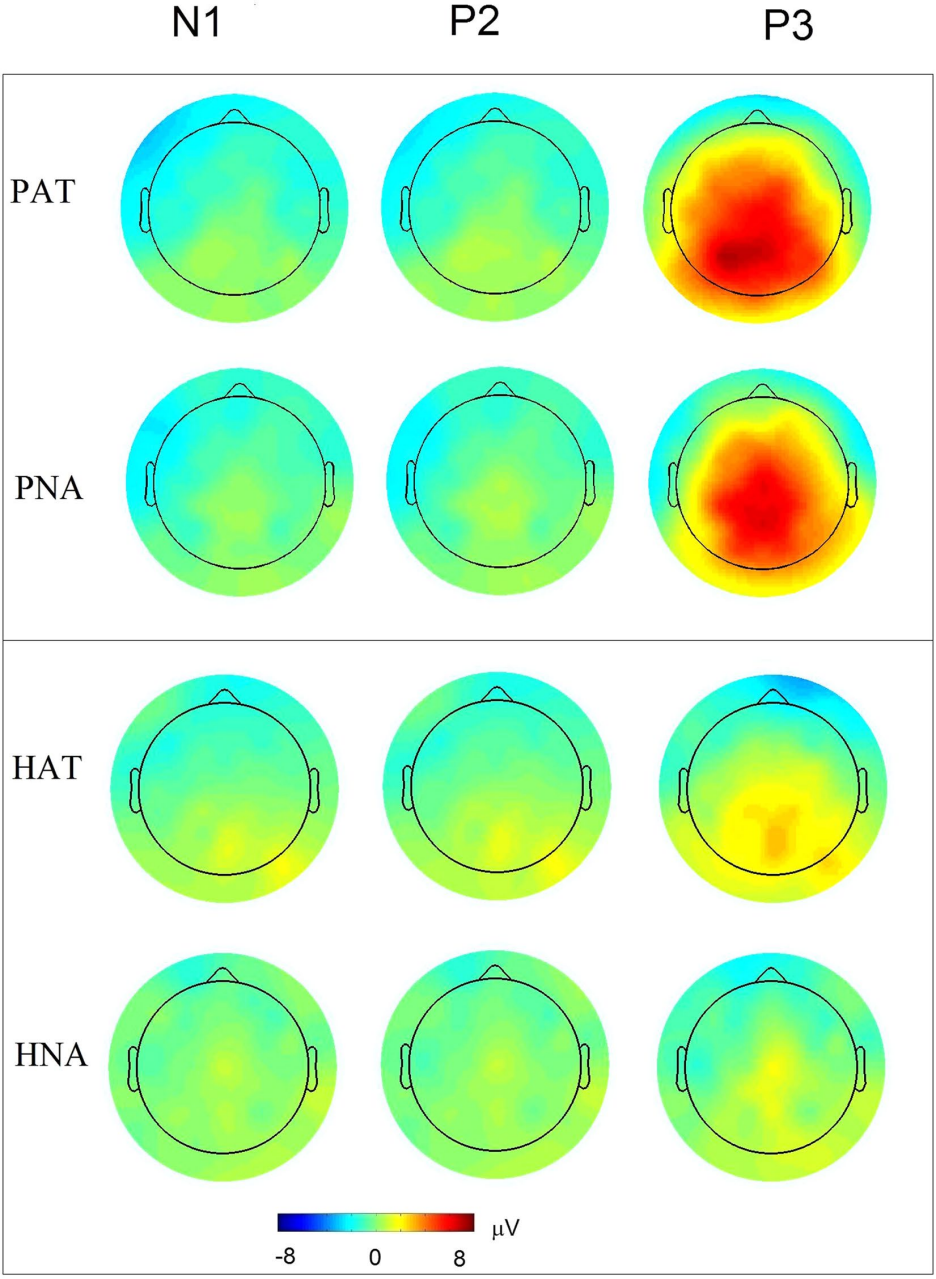
Topographical plots of N1, P2 and P3 components corresponding to PAT (PEA-AT), PNA (PEA-NA), HAT (H_2_O-AT), and HNA (H_2_O-NA). The odor conditions are presented in top and odorless in bottom panel, respectively.

For visual comparison, the ERP waveforms for all 64 electrodes pertaining to odor conditions, PAT and PNA, are shown in Fig. 2. The inset box illustrates N1, P2 and P3 peaks. The time intervals for N1, P2 and P3 were adjusted around their observed maxima on grand-averaged OERPs to 250–500 ms, 300–600 ms, and 650–1050 ms, respectively. The average OERP amplitudes corresponding to N1, P2 and P3 components for these conditions are plotted in a topographic map (Fig. 3).

**Figure 4:**
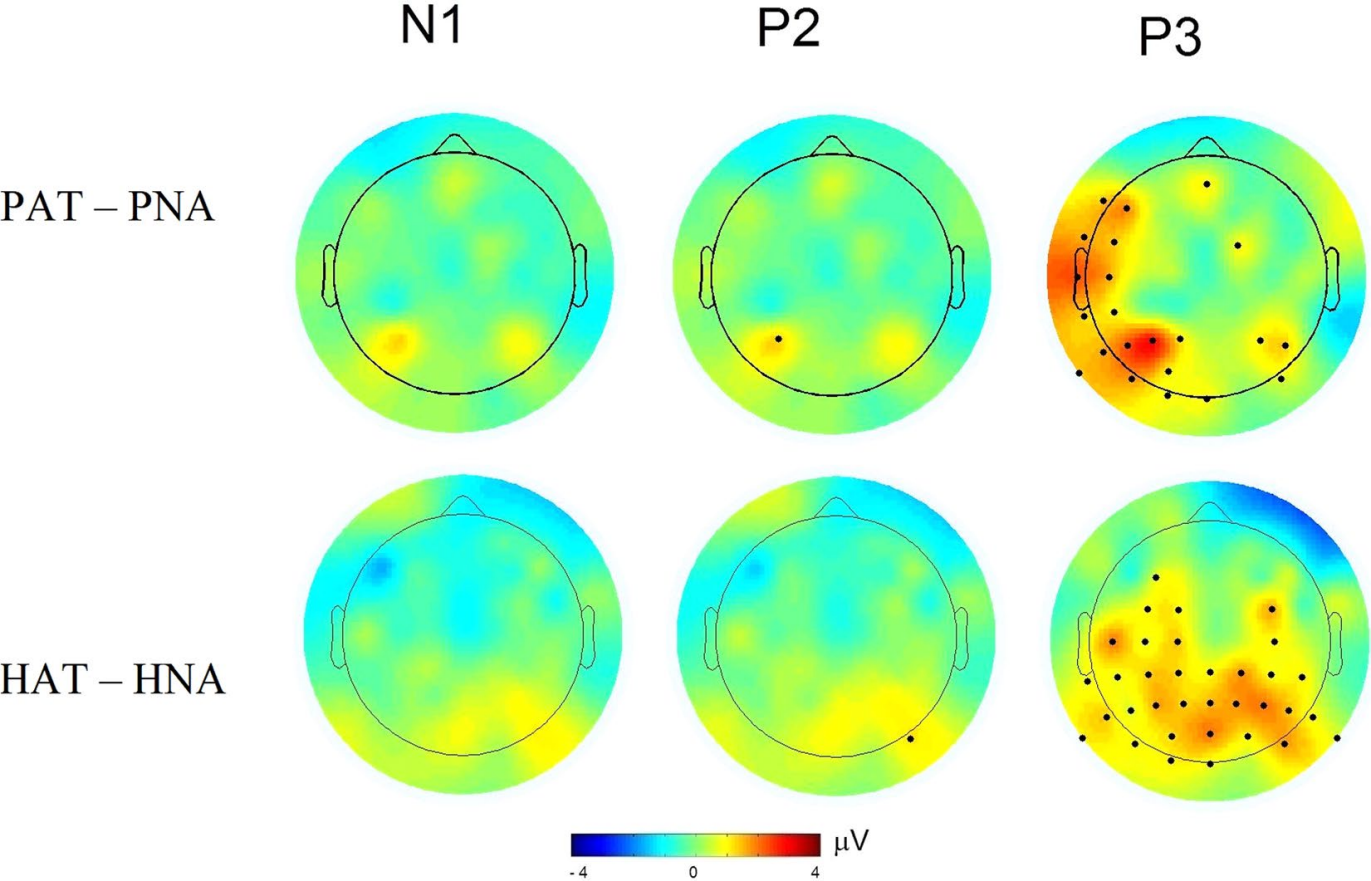
Topography of attentional modulation in N1, P2, P3 peak amplitudes. The top and bottom panel shows the amplitude differences between conditions of odor (PAT – PNA) and odorless (HAT – HNA) stimulus, respectively. The electrodes with the significant differences (fdr < 0.05) are marked in black dots (cf. Supplementary Tables S2 and S3).

Our main hypothesis was concerned with the task-related differences in amplitudes between PAT and PNA trial types. The bootstrap test (with 10000 replications) for amplitude difference showed significantly greater amplitude (fdr < 0.05) for PAT than PNA condition pertaining to P2 and P3 peak in the electrodes as marked (Fig. 4, top row). The statistics and q-values are listed in Supplementary Table S2. The topography of peak amplitude difference between PAT and PNA conditions is the most enhanced and significant for P3, as seen in 22 electrodes predominantly covering mid-frontal, left-frontal, left-temporal and bilateral parietal sites. There is one significant effect for P2 at electrode P3 (t = 1.2, q-value = 0.03), and no significant effect for N1 component. The bootstrap test for latency did not show significant difference between PAT and PNA conditions for any of the OERP components.

For OERP and topographic plots for filler stimulus conditions, VAT and VNA, refer to Supplementary Figs S3 and S4.

### Verification of OERP effects with odorless control ERP

The odorless stimulus served as a control. The ERP waveforms for all 64 electrodes pertaining to odorless conditions, HAT and HNA, are shown in Fig. 2. The average OERP amplitudes corresponding to N1, P2 and P3 components for these conditions are plotted in a topographic map (Fig. 3). Although the control stimulus, due to its lack of odor, is unlikely to show early N1 effect, it may show a late P2 or P3 effect reflecting the cognitive efforts made in searching for an odor, as observed in a slow rising potential 500 ms after the stimulus, peaking in the time interval of P3 component (Fig. 2). Nevertheless, this effect was found to be significantly lower compared to that of odor stimulus in both attend as well as not-attend conditions. The comparison between odor and control conditions (PAT vs HAT, and PNA vs HNA) using bootstrap test (10000 replications, fdr < 0.05) shows significantly greater P3 amplitude for the electrodes as marked in Supplementary Fig. S2.

In addition, the topographic maps of odorless contrast, HAT – HNA, are different from those of odor contrast, PAT – PNA (Fig. 4). The statistical comparison between HAT and HNA shows significant activation in 34 electrodes covering bilateral frontal, bilateral central and parietal scalp regions for P3, and one electrode, PO8, for P2 time intervals using bootstrap test (Fig. 4, bottom row).

### Source localization analysis

**Figure 5:**
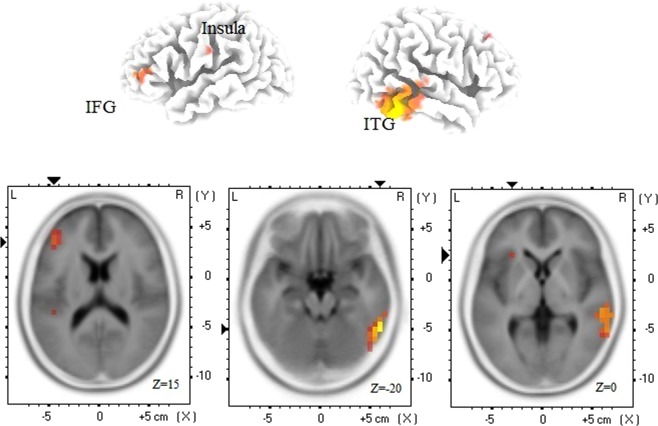
The sLORETA maps of odor contrast (PAT – PNA). The top panel shows the structures with larger PAT than PNA cortical current source density overlaid on rendered brain image using MNI template. Only the voxels that survived p-value threshold (P < 0.05, not corrected) are shown. The color represents t-value (red corresponding to low and yellow to high values). The columns in top panel represent left and right dorsal views. The bottom panel shows transverse slices for inferior frontal gyrus (IFG), inferior temporal gyrus (ITG), and insula. The voxel with significant peak activation (fdr < 0.05) in each of these structures is marked with a pair of black triangles. The statistics and p-values for these voxels are summarized in Table 1.

The cortical sources underlying P3 component that marked significant modulation from olfactory attention to PEA in OERP analysis were estimated with sLORETA. The voxels that showed greater activity for PAT than PNA were overlaid on the standard MNI brain template (Fig. 5) and summarized in Table 1. The structures with significant peak activation (fdr < 0.05) are distributed across frontal, parietal, temporal, and occipital lobes. In left frontal lobe, we see a cluster of activation surrounding inferior frontal gyrus overlapping with orbitofrontal gyrus. Parietal lobe includes activation surrounding inferior parietal lobe in postcentral gyrus. In insular cortex, the activation was significant in left hemisphere. The activation in temporal lobe was predominant in the right hemisphere encompassing inferior, middle, superior temporal, and fusiform gyri, extending to middle occipital gyrus.

**Table 1:** Summary of cortical sources for attentional modulation in olfactory P3 (PAT – PNA).

Lobe	H	Structure	BA	x	y	z	t-val	P	q-value
Frontal Lobe	L	Inferior Frontal Gyrus	46	−45	35	15	1.78	0.0070	0.0405
		Middle Frontal Gyrus	46	−45	45	10	1.85	0.0050	0.0338
	R	Middle Frontal Gyrus	8	30	35	45	1.61	0.0110	0.0490
Parietal Lobe	L	Postcentral Gyrus	1	−65	−20	30	1.61	0.0090	0.0449
Sub-lobar	L	Insula	13	−30	25	0	1.57	0.0110	0.0490
Occipital Lobe	R	Middle Occipital Gyrus	19	55	−60	−10	2.00	0.0030	0.0271
Temporal Lobe	R	Fusiform Gyrus	37	55	−60	−20	1.99	0.0030	0.0271
		Inferior Temporal Gyrus	20	60	−50	−20	2.28	0.0030	0.0271
		Middle Temporal Gyrus	20	60	−45	−15	2.27	0.0050	0.0338
		Superior Temporal Gyrus	22	60	−30	5	1.90	0.0050	0.0338

The brain structures shown in sLORETA maps (Fig. 5) are summarized based on the voxels with peak maxima. The summary provides the information on Lobe, Hemisphere (H), Structure, Brodmann Area (BA), the MNI co-ordinates (x, y, z), t-value (t-val), p-value (P), and q-value of the maxima in each structure. Only structures with at least three voxels are included. q-value represents an estimate of fdr-adjusted p-value (fdr < 0.05).

The corresponding results from sLORETA analysis for odorless H_2_O stimulus did not reveal any significant activation (fdr < 0.05).

## Discussion

In this study, we aimed to characterize the neural mechanisms underlying attentional modulation of olfactory processing using EEG recording. We examined olfactory attention by contrasting OERP data of attend (AT) with that of not-attend (NA) trials given an identical stimulus, PEA. Whereas AT trials required attention to odor for evaluating its pleasantness, NA trials did not require such attention. Our main hypothesis was that increased attentional load in AT would lead to greater peak amplitude and/or shorter peak latency than in NA, for P3 OERP component. We further hypothesized that the neuronal generators of such attentional modulation would underlie a network of brain areas that mediate olfactory attention. To verify this hypothesis, we referred to Royet *et al*.^18^, who examined the effect of attending (versus not attending) to odor pleasantness, and reported the involvement of orbitofrontal cortex, insula, precuneus, and middle temporal gyrus. Our results support both our hypotheses: (1) We found P3 to be the most dissociable biomarker of olfactory attention among all the OERP components. (2) The neural networks underlying the time interval of P3 component overlap with the hypothesized network, predominantly encompassing left inferior frontal gyrus (coinciding with orbitofrontal gyrus), left insula, and right temporal lobe.

Several studies in the past examined the effects of top-down olfactory attention on OERP measurements from three midline electrodes, using various experiment designs and stimuli^5–7,32^. Their designs involved odor detection or intensity evaluation task in attend and some non-olfactory distractor task (e.g., visual tracking) in not-attend trials. They used velopharyngeal closure breathing, which requires breathing through mouth. Although these experimental differences preclude a direct interpretation and comparison across studies, the evidence on P3 amplitude modulation from olfactory attention is consistent across studies including ours. In addition, our results present this evidence in both the spatial and temporal dimensions, revealing a characteristic topographical pattern with significant effects (Figs 3 and 4). We did not find significant P3 latency effect, as reported previously^4,6,7^. This disparity could possibly be attributed to the duration of stimulus evaluation, which is known to influence P3 latency^33,51^. The previous studies used immediate-response paradigm as opposed to delayed-response execution, and this may alter P3 latency. Another possibility is that the time required for evaluating olfactory pleasantness (used in our study) likely differs from that required for detection or intensity evaluation (used in previous studies).

Our results did not reveal any amplitude or latency modulation for N1 component. For P2 component, only amplitude modulation was observed at one electrode. Although they are generally associated with exogenous stimulus properties, they are also known to be influenced by top-down control of attention. For olfaction, such findings are scarce, e.g., previous OERP studies reported an attention induced latency reduction for P2^6^ and N1-P2^5^ components.

To get the OERP inferences as described above, we used conventional analysis that is based on averaging data around the concerned peaks estimated within a priori time intervals, which are quite well established for OERP. When the peak latencies are not certain, it is possible to examine the significance of difference between OERP components pertaining to two conditions at each time-point^52^.

The results from source localization analysis of the observed P3 effect point to a network of broadly distributed brain structures that revealed significantly larger current source density for PAT than PNA conditions (Fig. 5, Table 1). This activation network emerges in the brain maps corresponding to the post-stimulus time interval of P3 peak, 650–1050 ms, and reveals significant peak activation in the hypothesized brain regions as summarized below.

The frontal lobe reveals a cluster in inferior frontal gyrus, overlapping with lateral left orbitofrontal cortex. These are the most consistently reported regions for olfaction, especially for cognitively demanding olfactory tasks^12,53–56^. Previous psychological and neuroimaging studies suggested that olfactory pleasantness judgement involves odor identification^57,58^, and therefore may involve left orbitofrontal cortex close to language areas^18^.

The network also reveals significant activation in left insular cortex. The insula is frequently reported in functional neuroimaging research across various cognitive domains, and is known to have a role in integrating disparate function systems involving feelings, cognition, and action^59^. For olfaction, it is known to be involved in various odor processing tasks^15,19,60–64^, and specifically including tasks related to subjective olfactory pleasantness^15,16,18,56,63,64^.

The brain regions surrounding inferior parietal lobe also revealed significant activation. This region is known to have a role in attentive control on current task goals as well as responding to changes in stimuli in the environment^65,66^. We found additional significant activation in right temporal lobe, encompassing inferior, middle, and superior temporal, and fusiform gyri, which are associated with memory and emotions^18^. A couple of OERP studies have shown these structures among the cortical sources from a time period similar to the latency of P3 peaks for passive odor smelling^25^ and odor intensity rating^24^.

It is possible that AT involves enhanced vigilance and attention to the incoming air than NA trial type, regardless of whether it smells or not. Therefore, the brain regions identified by sLORETA analysis (PAT vs. PNA) of P3 component may reflect these and not olfactory attention per se. The observations from odorless control conditions rule out this possibility. Topographically, the effect of attending to odorless (versus odor) stimulus appears to be less evident showing significantly smaller P3 amplitudes for odorless than odor attend condition (PAT – HAT) for almost all electrodes (Supplementary Fig. S2, top row). The sLORETA analysis for odorless control stimulus did not reveal any activation network associated with significant larger neural activity in HAT than HNA condition from that for odor stimulus. This lends further support to our finding that the localized sources of attentional modulation in odor condition (as shown in Fig. 5) reflect attention to odor and not to the odorless stimulus.

This study has a limitation. Our experiment design includes only one odor stimuli, PEA. Therefore, we need to be cautious when extending our findings to other odors with different hedonic values. With the help of this design and methods, further research with new odors can extend our knowledge on how hedonic perception of an odor interacts with olfactory attention (See also Supplementary Discussion online). In addition, conscious olfactory processing as inherent in AT trial type may evoke other cognitive processes besides attention, such as emotion, decision making, or memory retrieval. Future investigations with new designs focusing on different odors and tasks would help in further dissociation among these cognitive factors.

In summary, we showed that P3 OERP components can effectively delineate olfactory attention for PEA odor in the post-stimulus interval of 650–1050 ms. Taken together with sLORETA-determined cortical current density in this time interval, our results suggest that olfactory attention may be mediated by a broad and distributed network of brain regions surrounding inferior frontal gyrus (coinciding with orbitofrontal gyrus), insula, and right temporal lobe. Our study illustrates a new approach of electrical neuroimaging using high-dimensional OERP measurements to provide both the temporal and cortical information of the neural dynamics underlying olfactory attention. We hope that these results will substantially contribute to the existing evidences from olfactory ERP/fMRI studies by adding the missing cortical/temporal dimension in their inference.

## Supplementary information

Supplementary Information
