# Supplementary material for: Electrophysiological correlates of top-down attentional modulation in olfaction
Source: Sci Rep. 2019 Mar 20;9:4953. doi: 10.1038/s41598-019-41319-6 (PMC6426950; doi:10.1038/s41598-019-41319-6)
Supplement: Supplementary file 1 — Supplementary Information [file 41598_2019_41319_MOESM1_ESM.pdf]

Supplementary Information

Electrophysiological correlates of top-down attentional modulation in olfaction

Archana K. Singh[1,2*], **Kazushige Touhara**[1,2], **and Masako Okamoto**[1,2*]

1 Department of Applied Biological Chemistry, Graduate School of Agricultural and Life Sciences, The University of Tokyo, Tokyo 113-8657, Japan
2 ERATO Touhara Chemosensory Signal Project, JST, The University of Tokyo, Tokyo 113-8657, Japan

*****sine.arc@gmail.com,
masakookamoto3@gmail.com

Supplementary Table S1: Open Essence scores for 31 Participants**.** The columns show subject-id, gender, open essence score, and age, respectively.

	**Gender**	**Open Essence Score**	**Age**
S1	F	11	33
S2	F	12	21
S3	M	8	22
S4	F	10	18
S5	M	10	20
S6	F	7	27
S7	M	10	22
S8	M	10	23
S9	M	10	22
S10	F	12	26
S11	M	12	21
S12	F	8	40
S13	F	11	38
S14	M	11	21
S15	F	11	22
S16	M	8	24
S17	M	9	26
S18	M	8	22
S19	M	10	20
S20	M	11	23
S21	F	11	23
S22	F	10	20
S23	M	11	22
S24	F	9	20
S25	M	10	20
S26	M	10	26
S27	M	10	20
S28	M	10	24
S29	M	9	21
S30	M	10	28
S31	F	11	21
	Median	10	22
	Mean	10	23.7
	SD	1.3	5.1

Supplementary Table S2: Statistics for electrodes with significant effects (fdr < 0.05), PAT – PNA corresponding to P3 OERP component. The columns represent, index, electrode label, observed t-statistic, and q-value, respectively.

	**Electrode**	**t**	**q-value**
1	F5	1.8376	0.0015
2	F7	1.4221	0.0019
3	FT7	1.5588	0.0013
4	FC5	1.3730	0.0048
5	C5	1.5683	0.0070
6	T7	2.2968	0.0008
7	TP7	1.2976	0.0044
8	CP5	0.9917	0.0250
9	P1	1.0833	0.0368
10	P3	2.8581	0.0013
11	P5	2.2532	0.0008
12	P7	1.6117	0.0012
13	P9	1.4661	0.0072
14	PO7	1.7236	0.0015
15	PO3	1.0720	0.0094
16	O1	0.8335	0.0464
17	Oz	0.8787	0.0233
18	AFz	0.9931	0.0410
19	FC2	0.8449	0.0390
20	P4	1.0522	0.0325
21	P6	1.3237	0.0064
22	PO8	0.6636	0.0406

Supplementary Table S3: Statistics for electrodes with significant effects (fdr < 0.05), HAT – HNA corresponding to P3 OERP component. The columns represent, index, electrode label, observed t-statistic, and q-value, respectively. For P2 OERP component, the significant effect was observed at only one electrode, PO8 (t = 1.4, q-value = 0.02)

	Electrode	t	q-value
1	F3	0.7130	0.0449
2	FC3	0.9893	0.0143
3	FC1	1.0159	0.0052
4	C1	1.3941	0.0002
5	C3	0.7718	0.0276
6	C5	1.8786	0.0002
7	TP7	0.8365	0.0231
8	CP5	0.8376	0.0151
9	CP3	1.3772	0.0006
10	CP1	1.2586	0.0128
11	P1	1.2955	0.0004
12	P3	1.6123	0.0004
13	P5	0.6622	0.0276
14	P7	1.3508	0.0067
15	P9	1.0622	0.0084
16	PO7	0.8906	0.0095
17	PO3	1.2825	0.0045
18	O1	1.0919	0.0116
19	Oz	0.8367	0.0093
20	POz	1.9349	0.0002
21	Pz	1.4810	0.0004
22	CPz	0.9749	0.0050
23	FC4	1.5979	0.0013
24	C4	0.9793	0.0045
25	CP6	1.0502	0.0145
26	CP4	0.8852	0.0232
27	CP2	1.7143	0.0002
28	P2	1.7266	0.0002
29	P4	1.9916	0.0002
30	P6	1.4348	0.0076
31	P8	0.8401	0.0212
32	P10	0.6245	0.0179
33	PO8	1.6561	0.0006
34	PO4	1.0806	0.0063

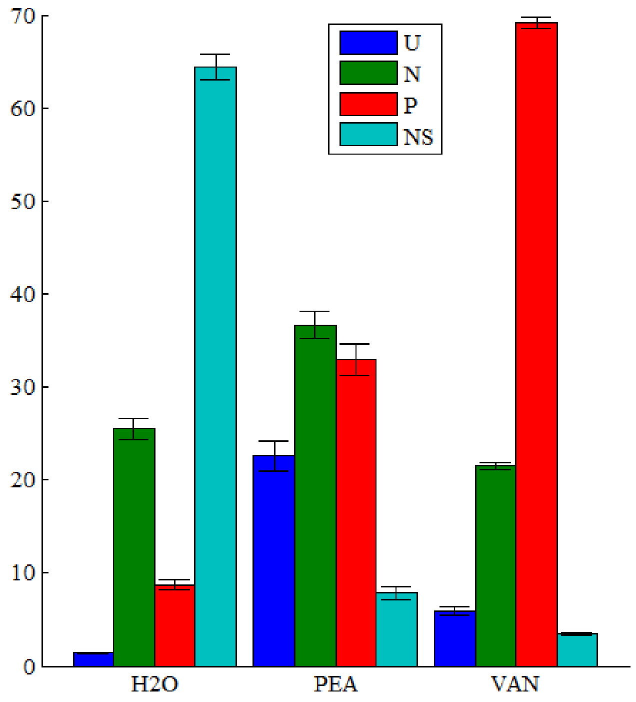

Supplementary Figure S1. The summary of pleasantness ratings obtained during EEG trials for HAT, PAT, and VAT conditions. The bars show Mean ± SD of the percentage of ratings obtained (N = 31). The ratings U, N, P, and NS represent 'Unpleasant', 'Neutral', 'Pleasant', and 'No Smell', respectively.

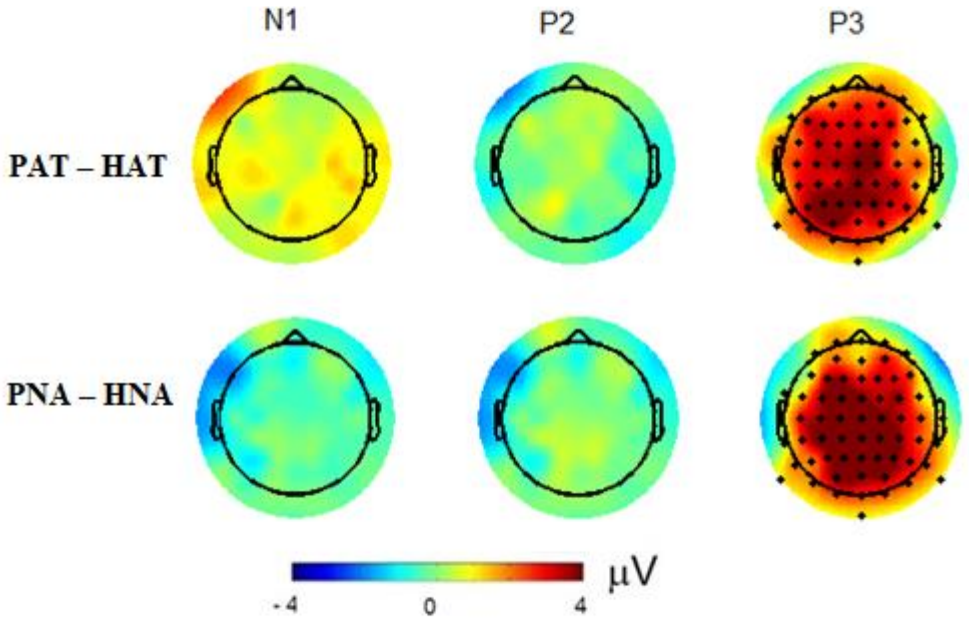

Supplementary Figure S2. Topography of difference in attentional modulation in N1, P2, P3 peak amplitudes: odor versus odorless. The top and bottom panel shows the amplitude differences in PAT versus HAT, PNA versus HNA, contrasts respectively. The electrodes with the significant differences are marked in black dots (fdr < 0.05).

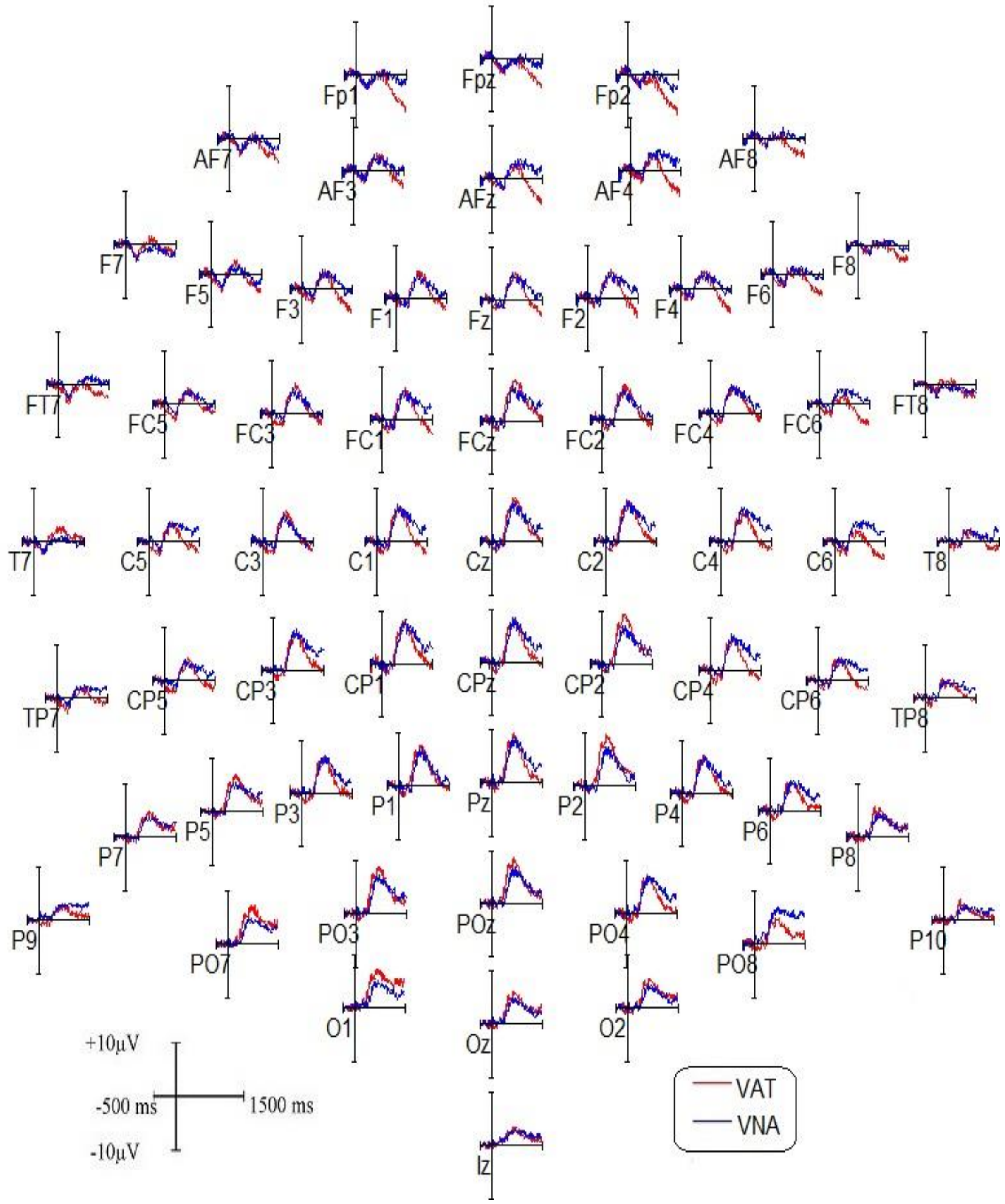

Supplementary Figure S3. O**ERP for VAN conditions.** ERP waveforms for VAN-AT and VAN-NA from 64 electrodes are shown in red and blue lines respectively.

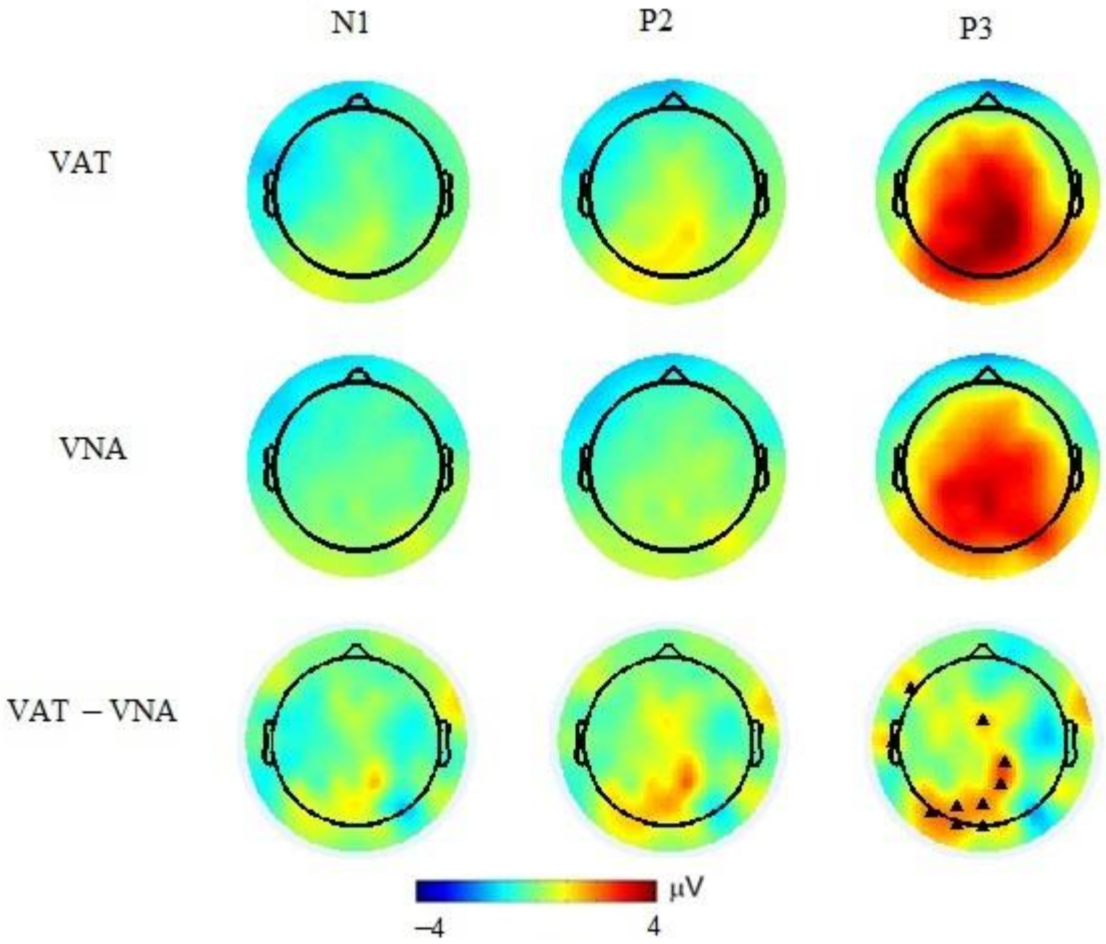

Supplementary Figure S4. **The topographic plots of VAN conditions.** The bottom most panel shows the amplitude differences (VAT − VNA); the electrodes with greater VAT than VNA amplitude (P<0.05, Uncorrected) are marked in black triangles for P3 OERP component.

Supplementary Discussion

Preliminary OERP Analysis with Vanillin stimulus

One limitation of this study is that it focuses only on single odor stimulus, PEA, which was found to be neutral by most participants. In response to a suggestion from one of the reviewers, we examined (from an exploratory aspect) the differences in top-down modulation effects with a hedonic odor stimulus such as VAN (rated as pleasant by most participants)

The VAN odor was included as filler stimulus in a few trials in between the main odor stimulus (PEA) and odorless control stimulus (H_2O) to prevent the predictability of main odor stimulus, which may create a potential response bias (Olofsson et al., 2014). Only few filler trials (12 each for VAT and VNA condition) were used to control response bias without lengthening EEG recording and adding subjects' fatigue. After removing noisy trials, the average number (Mean ± SD) of remaining trials was 10 ± 1 per condition, which is smaller than the number of trials recommended for a reliable statistical analysis (Hummel and Kobal, 2002).

The OERP waveforms show greater amplitude for VAT than VNA in middle and right central-parietal (CP2, P2 and POz), left frontal-temporal (F7, T7) and left parietal (P07, PO3 and O1) electrodes as shown by their waveforms in the time window of P3 component (Supplementary Fig. S3). The topographic patterns are shown in Supplementary Figure S4. The statistical test of VAT – VNA contrast did not show any significant electrodes (fdr < 0.05). Considering the exploratory purpose of this analysis, we marked the electrodes with greater VAT than VNA amplitude for P3 component using a more inclusive uncorrected p-value threshold, $P < 0.05$.

The effect in left side of the scalp (F7, T7, PO7, PO3 and O1) was also shown in the case of PEA (Fig.2 and Fig.3, main text), but the effect in the middle and right side of the scalp (CP2, P2 and POz) appears only for VAN. This difference in the topographic pattern of attentional modulation in olfaction may be attributed to the pleasantness, a stimulus-specific (bottom-up) factor, which was apparently greater in VAN than in PEA (Supplementary Figure S1).

However, as mentioned above, these findings are based on a preliminary analysis with different statistical threshold and stimulus frequency for VAN than PEA. Further experimentation with sufficient number of trials and more variety of odors will be helpful for a more objective examination and to provide a clearer perspective on how the hedonic perception of odors may interact with top-down attentional modulation that we observed in our study.

References:

1. Olofsson J.K., Time to smell: A cascade model of human olfactory perception based on response-time measurement. Front. Psychol. 5, 1–8 (2014)
2. Hummel, T., Kobal, G. Methods in Chemosensory Research (Frontiers in Neuroscience). Chapter 17, 429–444 (CRC Press, 2002)
